# The KIR2DL2/HLA-C1C1 Gene Pairing Is Associated With an Increased Risk of SARS-CoV-2 Infection

**DOI:** 10.3389/fimmu.2022.919110

**Published:** 2022-07-07

**Authors:** Song Hu, Zuoyu Shao, Wei Ni, Pan Sun, Jialu Qiao, Hexing Wan, Yi Huang, Xiaolong Liu, Haoyang Zhai, Mingzhong Xiao, Binlian Sun

**Affiliations:** ^1^ Wuhan Institute of Biomedical Sciences, School of Medicine, Jianghan University, Wuhan, China; ^2^ Hepatic Disease Institute, Hubei Key Laboratory of Theoretical and Applied Research of Liver and Kidney in Traditional Chinese Medicine, Hubei Provincial Hospital of Traditional Chinese Medicine, Wuhan, China; ^3^ Hepatic Disease Institute, Hubei Province Academy of Traditional Chinese Medicine, Wuhan, China

**Keywords:** COVID-19, SARS-CoV-2, NK cells, HLA, KIR

## Abstract

SARS-CoV-2 is the causative agent for the global COVID-19 pandemic; however, the interaction between virus and host is not well characterized. Natural killer cells play a key role in the early phase of the antiviral response, and their primary functions are dependent on signaling through the killer cell immunoglobulin-like receptor (KIR). This study measured the association between KIR/HLA class I ligand pairings and the occurrence and development of COVID-19. DNA of blood samples from 257 COVID-19 patients were extracted and used to detect KIR and HLA-C gene frequencies using single strain sequence-specific primer (SSP) PCR. The frequency of these genes was compared among 158 individuals with mild COVID-19, 99 with severe disease, and 98 healthy controls. The frequencies of KIR2DL2 (P=0.04, OR=1.707), KIR2DS3 (P=0.047, OR=1.679), HLA-C1C1 (P<0.001, OR=3.074) and the KIR2DL2/HLA-C1C1 pairing (P=0.038, OR=2.126) were significantly higher in the COVID-19 patients than the healthy controls. At the same time, the frequency of KIR2DL3+KIR2DL2-/HLA-C1+Others+ was lower in COVID-19 patients than in healthy individuals (P=0.004, OR=0.477). These results suggest that the protective effect of KIR2DL3 against SARS-CoV-2 infection is related to the absence of the KIR2DL2 gene. This study found no correlation between the frequencies of these genes and COVID-19 pathogenesis. Global statistical analysis revealed that the incidence of COVID-19 infection was higher in geographic regions with a high frequency of KIR2DL2. Together these results suggest that the KIR2DL2/HLA-C1C1 gene pairing may be a risk factor for SARS-CoV-2 infection.

## Introduction

On January 30, 2020, the World Health Organization (WHO) declared the 2019 Coronavirus Disease (COVID-19) outbreak an important public health event (1). Severe acute respiratory syndrome coronavirus 2 (SARS-CoV-2), a coronaviruses family member, is characterized by a single-stranded RNA with positive sense. The coronavirus genome is highly susceptible to mutations, resulting in genetic drift and allowing the virus to evade immune recognition (2). The virus is still circulating worldwide, and the new coronavirus disease caused by SARS-CoV-2 is a public health crisis that requires an active response (3). Studies of SARS-CoV-2 infection have shown that this infection spreads easily among immediate family members. The speed of viral transmission and the pathogenicity of this disease differ by geographic region and while this is related to differences in the implementation of prevention and control strategies, the occurrence and progression of COVID-19 may also be related to the host’s genetic background (4).

Human leukocyte antigen (HLA) polymorphism is a genetic factor associated with variations in susceptibility to virus infection. For example, SARS-CoV-2 patients in Saudi Arabia were found to have a higher frequency of HLA-A*01, B*56, and C*01 than uninfected individuals, and the frequency of HLA-A*03 and C*06 was particularly high in patients who died of COVID-19 (5). In a Japanese study, HLA-A*11, HLA-C*12, and HLA-B*52 correlated significantly with disease severity (6). In Italy, COVID-19 infection is typically more severe in the northern region and milder in the southern region. The regional distribution of HLA-C*07 in the Italian population is positively correlated with COVID-19 morbidity and mortality (7). SARS-CoV-2 infection disrupts immune homeostasis, leading to an imbalance of the immune regulatory system. Infection can also result in a decline in macrophages and NK cells (8–10).

NK cells are important lymphocytes of the innate immune response that can directly kill target cells without damaging healthy cells (11). This cell type is relatively abundant, accounting for 5–20% of lymphocytes (12), and plays a key role in both antiviral and antitumor immunity. NK cell activation is dependent on signals triggered by an array of inhibitory and activating receptors that ensure self-tolerance against healthy cells while destroying virally infected target cells. The killer cell immunoglobulin-like receptor (KIR), an immunoglobulin superfamily member, is one of the important surface receptors involved in NK cell activation. KIRs are expressed on the surface of NK cells and some T lymphocytes (13). NK cells account for about 15% of cells in the human lung and CD56^dim^ NK cell subsets expressing KIR and other receptors account for 60–80% of this population (14). To date, 16 distinct KIR gene loci, including the KIR2DP1 and KIR3DP1 pseudogenes, have been identified, which has created considerable diversity in the number and type of KIR expressed on the NK cell surface. Many KIR ligands have been identified, including KIR3DL1 combined with HLA-Bw4 (15), KIR2DL1 combined with HLA-Cw2, HLA-Cw4, HLA-Cw5 or HLA-Cw6 (called C2), and KIR2DL2, KIR2DL3 and KIR2DS2 combined with HLA-Cw1, HLA-Cw3, HLA-Cw7 or HLA-Cw8 (called C1) (16). Based on the polymorphisms at positions 77 and 80 in the HLA heavy chain a1 domain, KIR2DL1, KIR2DL2/3, and KIR2DS2 recognize different HLA-C allotypes. In Caucasians, patients with persistent hepatitis C virus (HCV) infection have a higher frequency of KIR2DL3 than those who have successfully cleared infection (17). KIR2DL3 is also closely related to the occurrence of fatal cerebral malaria (18) and KIR3DS1 is associated with the delayed development of AIDS and clearance of HCV (19).

The individual immune response is closely related to the KIR/HLA-C pairing. At the same time, viral replication and pathogenicity are associated with the genetic background of the host. This study assessed the frequency of the KIR and HLA-C genes in COVID-19 patients infected in the early phase of the pandemic and healthy people living in Wuhan, China, and identified specific protective or susceptible KIR/HLA-C gene pairings.

## Materials and Methods

### Study Subjects

This study conformed to the 1975 Declaration of Helsinki guidelines and permission was obtained from the Ethics Committee of Jianghan University and Hubei Provincial Hospital of Traditional Chinese Medicine. From January to October 2020, the research team recruited 98 healthy volunteers who were not infected with SARS-CoV-2 and 257 COVID-19 patients who received rehabilitation treatment at the Hubei Provincial Hospital of Traditional Chinese Medicine in Wuhan, China to conduct a retrospective study. All participants were Chinese Han.

COVID-19 patients were divided into a group of 158 individuals with mild infection and a group of 99 people with severe infection using the Guidelines on the Diagnosis and Treatment of Novel Coronavirus issued by the National Health Commission in China. In brief, mild disease was defined as a lack of respiratory symptoms and no pulmonary radiological manifestations, or mild respiratory symptoms and radiological evidence of pneumonia. Severe cases were defined as SpO2 ≤93% that required oxygen support or requiring heart-lung machine support for acute respiratory distress syndrome. All study participants were aware of the research content and met the ethical requirements.

### DNA Extraction and Genotyping

Genomic DNA was extracted from 5 ml EDTA anticoagulated peripheral blood using an SE Blood DNA Kit (Omega Bio-Tek Inc, USA). Fifty forward and reverse primer pairs were used to specifically amplify the KIR gene by sequence-specific primer (SSP) PCR (20). SSP PCR was performed according to a method based on the HLA-C allele that was described previously (21). After separation on a 1.5% agarose gel, it was determined whether there was a PCR product. All gels were assessed by two independent observers using a DNA gel imaging system and if any inconsistencies arose, the samples were re-typed.

### Statistical Methods

Using SPSS 18.0 software for Windows, KIR/HLA genotype frequencies were compared between healthy controls and COVID-19 patients using the Chi-square test, p-values, and Odds ratios (ORs). ORs and 95% confidence intervals (CIs) were calculated using the quantitative data from COVID-19 patients and healthy controls. The 95% CI confidence interval for a meaningful OR value excludes the value of 1. The test data is only considered statistically significant if both the p-value and the OR value of the gene or paired combination frequency are statistically significant. The same method was used to analyze the frequencies of related genes in COVID-19 patients with mild and severe disease.

## Results

### Participant Recruitment and Testing

The study subjects were all from the Hubei Provincial Hospital of Traditional Chinese Medicine. Ninety-eight healthy volunteers (59 males and 39 females) were verified as being COVID-19 negative using nucleic acid testing and medical history review and had no history of HIV, HBV, or HCV infection. All volunteers lived in Wuhan during the COVID-19 outbreak. The 257 COVID-19 patients included 158 (69 men and 89 women) with mild disease and 99 (49 men and 50 women) with severe disease who were diagnosed with COVID-19 using a nucleic acid test, CT scan, and other routine tests. After recovery, the patients received rehabilitation treatment at the Hubei Provincial Hospital of Traditional Chinese Medicine (**Table 1**).

**Table 1:** Clinical characteristics and the frequencies of KIR2DL2, KIR2DS3, and HLA-C1 in COVID-19 patients and healthy persons.

Characteristic	COVID-19 Patients	Health Controls	P Value	Odss Ratio
	(n=257) (%)	(n=98) (%)		(95% CI)
Sex (M:F)	118:139	59:39		
Mean age (span; median)	(25-80); 55.5	(19-69); 33.8		
KIR2DL2	98 (38.13)	26 (26.53)	0.040	1.707 (1.020-2.854)
KIR2DS3	97 (37.74)	26 (26.53)	0.047	1.679 (1.004-2.808)
KIR2DL2+/2DS3+	56 (21.79)	12(12.25)	0.041	1.997 (1.019-3.912)
HLA-C				
CI+/CI+	121(47.08)	22 (22.45)	<0.001	3.074 (1.802-5.243)
CI+/C2+	25 (9.73)	14 (14.29)	0.220	0.647 (0.321-1.302)
CI+/Others+	80 (31.13)	42 (42.86)	0.038	0.603 (0.373-0.973)
C2+/C2+	6 (2.33)	1 (1.02)	0.262	2.319 (0.276-19.51)

### KIR2DL2, KIR2DS3, and HLA-C1 Are Genetic Risk Factors for COVID-19 Infection

KIR gene frequency was compared between COVID-19 patients and healthy controls (**Table 1**). KIR2DL2 and KIR2DS3 were more highly frequent in infected than uninfected individuals (KIR2DL2: 38.13% vs 26.53%, respectively; OR=1.707, P=0.04; KIR2DS3: 37.74% vs 26.53%, OR=1.689, P=0.047). The frequency of KIR2DL2+/KIR2DS3+ was also higher in COVID-19 patients than healthy controls (21.79% vs 12.25%, respectively; OR=1.997, P=0.041, 95% CI=1.019–3.912). These data suggest that KIR2DL2 and KIR2DS3 may be genetic risk factors for SARS-CoV-2 infection.

KIR activity requires the participation of HLA ligands. The frequency of HLA-C1C1, the homozygous receptor for KIR2DL2 and KIR2DL3, was also higher in COVID-19 patients than in healthy controls (47.08% vs 22.45%, respectively; OR=3.074, P <0.001). It is suggested that the HLA-C1C1 gene pairing is a risk factor for COVID-19 (**Table 1**). Further analysis indicated that the frequency of KIR2DL2/HLA-C1+C1+ was higher in the COVID-19 patients than in the healthy controls (19.46% vs 10.2%, respectively; OR=2.126, P=0.038). These findings suggest that the KIR2DL2/HLA-C pairing is a genetic risk factor for COVID-19 (**Table 2**).

**Table 2:** Pairing analysis of the frequencies of KIR2DL2/2DL3/2DS2 and HLA-C in COVID-19 patients and healthy persons.

Characteristic		COVID-19 Patients	Health Controls	P Value	Odss Ratio
		(n=257) (%)	(n=98) (%)		(95% CI)
KIR2DL2/HLA-C					
	SDL2+/CI+CI+	50 (19.46)	10 (10.20)	0.038	2.126 (1.031-4.381)
	2DL2+/CI+C2+	14 (5.45)	6 (6.12)	0.805	0.883 (0.330-2.368)
	2DL2+/CI+Others	28 (10.90)	8 (8.16)	0.446	1.376 (0.604-3.132)
KIR2DL3/HLA-C					
	2DL3+/CI+CI+	120 (46.69)	22 (22.45)	<0.001	3.026 (1.774-5.162)
	2DL3+/CI+C2+	26 (10.12)	14 (14.29)	0.267	0.675 (0.337-1.355)
	2DL3+/CI+Others	79 (30.74)	41 (41.84)	0.048	0.617 (0.381-0.998)
					
	2DL3+2DL2-/CI+Others+	52 (22.23)	34 (34.69)	0.004	0.477 (0.285-0.799)
	2DL3+2DL2+/CI+Others+	17 (6.61)	7 (7.14)	0.859	0.921 (0.370-2.294)
KIR2DL2/HLA-C					
	2DS2+/CI+CI+	61 (23.74)	11 (11.22)	0.009	2.462 (1.235-4.907)
	2DS2+/CI+C2+	15 (5.84)	7 (7.14)	0.648	0.806 (0.318-2.040)
	2DS2+/CI+Other+	30 (11.67)	18 (18.37)	0.099	0.587 (0.310-1.111)

### The KIR2DL3/HLA-C1 Pairing May be a Genetic Protective Factor Against COVID-19 Infection

Assessment of HLA-C distribution among the study subjects revealed a combination of HLA-C alleles in which one locus was C1 and the other locus was neither C1 nor C2. We termed this ‘Other’ or ‘O’ and named the combination HLA-C1+O+. The frequency of HLA-C1+O+ was lower among COVID-19 patients than healthy controls (31.13% vs 42.86%, respectively; OR = 0.603, P=0.038). Thus, it is possible that the HLA-C1+O+ combination is a genetic protective factor against SARS-CoV-2 infection (**Table 2**). Similarly, the KIR2DL3/HLA-C1+O+ combination was lower in the COVID-19 patients than in healthy individuals (30.74% vs 41.84%, respectively; OR =0.617, P= 0.048). To determine whether KIR2DL2 also plays a protection function, individuals with KIR2DL3/HLA-C1+O+ were divided into two groups based on their exist of KIR2DL2. COVID-19 patients were still less likely to carry KIR2DL3+KIR2DL2-/HLA-C1+O+ than healthy controls (22.23% vs 34.69%, respectively, OR =0.477, P=0.004), suggesting that this genetic combination is still protective in the absence of KIR2DL2 (**Table 2**). In contrast, the frequency of KIR2DL3+KIR2DL2+/HLA-C1+O+ was similar between COVID-19 patients and controls (6.61% vs 7.14%, respectively; OR =1.086, P=0.859). These results show that the protective effect of the KIR2DL3/HLA-C1 pairing is dependent on the absence of KIR2DL2.

### KIR2DL2 Distribution Correlates Positively With the COVID-19 Caseload in Different Geographic Regions

**Figure 1:**
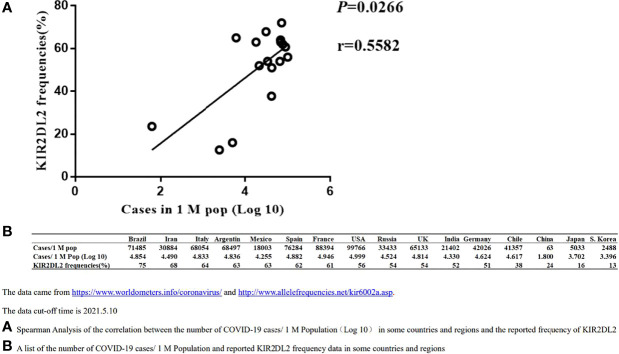
Spearman Correlation analysis between the distribution of KIR2DL2 and the occurrences of global COVID-19 cases. The data cut-off time is 2021.5.10. **(A)**. Spearman Analysis of the correlation between the number of COVID-19 cases/1 M Population(Log 10) in some countries and regions and the reported frequency of KIR2DL2. **(B)**. A list of the number of COVID-19 cases/1 M Population and reported KIR2DL2 frequency data in some countries and regions.

To confirm the association between KIR2DL2 distribution and SARS-CoV-2 infection, we used public information platforms (https://www.worldometers.info/coronavirus/) to obtain the incidence of COVID-19 (Case/1 M pop) in different geographic regions as of May 10, 2021 (**Figure 1**).

The following equation was used to calculate incidence:

Case/1 M pop = total number of COVID-19 cases/population of region*1,000,000

The frequency of KIR2DL2 in geographic regions with available public data was determined using relevant websites (http://www.allelefrequencies.net/kir6002a.asp), and the frequency of KIR2DL2 reported in each region was used for follow-up analysis. Spearman correlation analysis suggested that KIR2DL2 distribution was positively correlated with COVID-19 incidence (p=0.0266, r=0.5582) (**Figure 1**).

### COVID-19 Severity May Not Correlate Directly With KIR Gene

To determine if KIR gene was associated with COVID-19 severity, the same combinations of KIR2DL2, KIR2DS3/HLA-C, and KIR2DL3/HLA-C were compared between patients with mild and severe disease. There were no significant differences in disease severity, suggesting that the KIR gene may not correlate with COVID-19 pathogenesis (**Table 3**).

**Table 3:** Correlation analysis of KIR/HLA-C between mild and severe COVID-19 patients.

Characteristic		COVID-19 Patients	Health Controls	P Value	Odss Ratio
		(n=257) (%)	(n=98) (%)		(95% CI)
Sex(M:F)		69:89	49:50:00		
Mean age (span, median)		(29-79); 55.4	(32.80); 55.7		
KIR2DL2		57 (36.08)	41 (41.41)	0.391	0.798 (0.477-1.336)
KIR2DS3		61 (38.61)	35 (35.35)	0.600	1.150 (0.682-1.938)
KIR2DL2+/2DS3+		34(21.52)	17 (17.17)	0.395	1.323 (0.694-2.522)
HLA-C1	CI+/CI+	72 (45.57)	48 (49.49)	0.716	0.911 (0.550-1.508)
	CI+/C2+	17 (10.76)	9 (9.09)	0.666	1.206 (0.515-2.821)
	CI+/Others+	48 (30.38)	32 (32.32)	0.743	0.914 (0.532-1.569)
	C2+/C2+	4 (2.53)	2 (2.02)	0.792	1.260 (0.226-7.009)
KIR2DL2/HLA-C	2DL2+/CI+CI+	27 (17.09)	23 (23.23)	0.226	0.681 (0.365-1.271)
	2DL2+/CI+C2+	9 (5.69)	5 (5.05)	0.824	1.136 (0.369-3.192)
	2DL2+/CI+Others+	16 (10.13)	12 (12.12)	0.617	0.817 (0.369-1.569)
KIR2DL3/HLA-C	2DL3+/CI+CI+	71 (44.94)	49 (49.49)	0.476	0.833 (0.503-1.378)
	2DL3+/CI+C2	17 (10.76 )	9 (9.09)	0.666	1.206 (0.537-1.875)
	2DL3+/CI+Others	48 (30.38)	32 (32.32)	0.743	0.914 (0.532-1.569)
	2DL3+2DL2-/CI+ Others+	32 (20.25)	20 (20.20)	0.992	1.003 (0.537-1.875)
	2DL3+2DL2+/CI+ Others+	16 (10.13)	12 (12.12)	0.617	0.817 (0.369-1808)
KIR2DS2/HLA-C					
	2DS2+/CI+CI+	38 (24.05)	23 (23.23)	0.881	1.046 (0.579-1.892)
	2DS2+/CI+C2+	11 (6.96)	4 (4.04)	0.331	1.777 (0.550-5.744)
	2DS2+/CI+Others	18 911.39)	12 (12.12)	0.859	0.932 (0.428-2.029)

## Discussion

The occurrence of viral infections is shown to be closely related to an individual’s genetic background (22–24). NK cells are fast-acting innate immune cells that provide a critical first line of defense by killing infected cells and producing pro-inflammatory cytokines (25). The current study found that KIR2DL2 gene frequency was much higher in COVID-19 patients than in healthy controls. Interestingly, while there were no differences in KIR2DL3 in the initial analysis, this gene was found to have a protective effect when combined with HLA-C1+O+ in the absence of KIR2DL2. Indeed, the KIR2DL2/HLA-C1+C1+ pairing appeared more frequently in COVID-19 patients than in healthy individuals. We also analyzed current public data and found that the frequency of KIR2DL2 in the population correlated positively with the number of COVID-19 cases in particular geographic regions. These results suggest that people carrying the KIR2DL2/HLA-C1C1 gene may be at high risk for COVID-19. KIR2DL2 is known to inhibit NK activation, which may prevent the early clearance of SARS-CoV-2.

KIR plays an important role in aiding the immune response against virus-infected cells. KIR3DL1 is more highly frequent in HBV-infected individuals (26) and KIR2DS1 may reduce the risk of CMV infection after kidney transplantation (27). Our previous study found that the presence of KIR2DL2 inhibits the NK cell response to HCV-infected patients in the Chinese Han population. Indeed, KIR2DL2 is not only associated with the HCV infection rate but also with HCV clearance (28). These results suggest that KIRs play a role in the occurrence and persistence of infectious diseases.

The KIR2DL2 and KIR2DL3 cDNA sequences have been given different names, but genome analysis and population studies indicate that they may be alleles at the same locus (29), and share high homology. KIR2DL2 combines more strongly with HLA-C than KIR2DL3 (30) and may exert a more powerful inhibitory effect on NK cells that promotes survival of the virus. KIR expression on NK cells is largely random (31), so it is possible that some people carrying the KIR2DL2 gene may have more KIR2DL2+CD56^dim^NK cell subgroups that weaken the antiviral response and promote infection. KIR2DL3+ also plays inhibitory function but is weaker than KIR2DL2+, so KIR2DL3+ NK cells are more easily activated, which might inhibit the virus at the early stage of infection to prevent disease.

Our data implied that KIR/HLA-C is not related to the progression of COVID-19. One possible explanation is that CD4+ and CD8+ T lymphocytes are the main cells involved in fighting this disease, and contribute to the development of severe outcomes such as the “cytokine storm” (32). KIR is mainly expressed on the surface of CD56^dim^ NK cells. This NK cell subtype is primarily involved in killing infected target cells, and cytokine secretion is limited (33). It is also possible that an association between KIR/HLA-C and COVID-19 progression was not observed because this study was primarily limited to the Chinese Han population. In a recent study of 424 COVID-19 positive patients (392 Saudi, 32 non-Saudi), the KIR2DS4 gene was associated with the highest risk of severe COVID-19 infection (OR 8.48, p= 0.0084) followed by KIR3DL1 (OR 7.61, p=0.0192) (34). The absence of KIR3DL1+HLA-Bw4+ and KIR3DL2+HLA-A3/11+ pairings were protective against COVID-19 progression in 200 hospitalized patients at the University of California San Francisco Medical Center during March–October 2020 (35). KIR genotypes vary widely across ethnic groups (36). All of these research suggested that KIR genes involved in COVID-19 progression, and different KIR gene might play different roles.

This study provides a correlation between the immune genetic background of some Han people and COVID-19. In particular, KIR2DL2/HLA-C1+C1+ may be a risk factor for SARS-CoV-2 infection in this population. KIR are a group of molecules that play an important role in regulating the differentiation, development, and activation of NK cells. The random expression of KIR genes among individuals may change the distribution of disease in different populations. The current study is primarily focused on gene frequency and disease. Further research will be required to characterize the mechanism of KIR expression, such as epigenetic regulation, in the occurrence and development of infectious diseases. This will help to inform our understanding of host–virus interactions.

## Data Availability Statement

The original contributions presented in the study are included in the article/supplementary material. Further inquiries can be directed to the corresponding authors.

## Ethics Statement

The project was approved after discussion by the Ethics Committee of Hubei Province Academy of Traditional Chinese Medicine (HBZY2020-CO1-01). The patients/participants provided their written informed consent to participate in this study.

## Author Contributions

BS and SH jointly completed the experimental design and article writing. ZS and MX were mainly involved in the recruitment of COVID-19 survivors and the division of experimental cohorts. WN and PS were responsible for the collection and sorting of peripheral blood of COVID-19 survivors. HW, YH, and XL were mainly involved in PCR-SSP and result reading and recording. JQ focused on the quality control and analysis of results. All authors contributed to the article and approved the submitted version.

## Funding

This research was funded by Wuhan Municipal Health Commission (No. EX20B02) and Jianghan University Funds (3015/06210035, 1010/08190006).

## Conflict of Interest

The authors declare that the research was conducted in the absence of any commercial or financial relationships that could be construed as a potential conflict of interest.

## Publisher’s Note

All claims expressed in this article are solely those of the authors and do not necessarily represent those of their affiliated organizations, or those of the publisher, the editors and the reviewers. Any product that may be evaluated in this article, or claim that may be made by its manufacturer, is not guaranteed or endorsed by the publisher.

## Abbreviations

COVID-19, 2019 Coronavirus Disease; SARS-CoV-2, Severe acute respiratory syndrome coronavirus 2; NK cells, Natural killer cells; HLA, Human leukocyte antigen; KIR, Killer cell immunoglobulin-like receptor.
